# Link Prediction Investigation of Dynamic Information Flow in Epilepsy

**DOI:** 10.1155/2018/8102597

**Published:** 2018-07-02

**Authors:** Yan He, Fan Yang, Yunli Yu, Celso Grebogi

**Affiliations:** ^1^School of Biology and Engineering, Guizhou Medical University, Guiyang, Guizhou Province, China; ^2^Department of Neurology, Affiliated Hospital of Guizhou Medical University, Guiyang, Guizhou Province, China; ^3^Institute for Complex Systems and Mathematical Biology, King's College, University of Aberdeen, Aberdeen AB24 3UE, UK

## Abstract

As a brain disorder, epilepsy is characterized with abnormal hypersynchronous neural firings. It is known that seizures initiate and propagate in different brain regions. Long-term intracranial multichannel electroencephalography (EEG) reflects broadband ictal activity under seizure occurrence. Network-based techniques are efficient in discovering brain dynamics and offering finger-print features for specific individuals. In this study, we adopt link prediction for proposing a novel workflow aiming to quantify seizure dynamics and uncover pathological mechanisms of epilepsy. A dataset of EEG signals was enrolled that recorded from 8 patients with 3 different types of pharmocoresistant focal epilepsy. Weighted networks are obtained from phase locking value (PLV) in subband EEG oscillations. Common neighbor (CN), resource allocation (RA), Adamic-Adar (AA), and Sorenson algorithms are brought in for link prediction performance comparison. Results demonstrate that RA outperforms its rivals. Similarity, matrix was produced from the RA technique performing on EEG networks later. Nodes are gathered to form sequences by selecting the ones with the highest similarity. It is demonstrated that variations are in accordance with seizure attack in node sequences of gamma band EEG oscillations. What is more, variations in node sequences monitor the total seizure journey including its initiation and termination.

## 1. Introduction

Link prediction works on revealing edge production based on network topology and node attributes. Meanwhile, higher prediction capability corresponds to more accurate description of network evolution, and it has been successfully adopted in social networks such as recommendation system. However, it is unclear that link prediction could be adopted for uncovering brain dynamics.

Brain is a complex system where multiple components work together for cognitive function. Brain is so fragile that it could be damaged forever after occasional trauma [1]. The cooperation and synergy among cerebral areas are fundamental for capability maintenance which could be investigated by graph theory as a network [2, 3]. When brain is taken as a network, individual brain areas are considered as nodes and their interaction are links. The penetration of complex network for brain research has become popular and effective. Brain connectome reflects individual characteristics, and these wiring patterns could be taken as fingerprint of people [4]. Statistical comparison of network parameters is an objective description of brain disorders. Time-varying network changes reveal spatiotemporal alterations in the brain [5]. Certain structure affects network function, and small-worldness topology refers to quick information integration and requires neural remodeling [6]. As a cooperative element, pathological condition might stem from disconnection or rearrangement in the brain [7–9]. Investigation of pathological brain architecture changes aids in not only clinical diagnosis and early warning, but also mechanism discovery. However, more subjective description techniques are needed for investigation of evolutionary process, especially of brain progressions under pathological condition.

As a common brain disorder, epilepsy affects more than 60 million people around the world, and one third of them are refractory to medicine which require precise diagnosis and risk assessment during daily life. High amplitude synchronization is an obvious signal of epilepsy under seizure attack [10]. Multichannel EEG data records brain function under various conditions [11]. It has become indispensable in clinical diagnosis for epilepsy as an efficient mirror of neural behaviors of the brain. Detailed description of EEG signals aids in clinical evaluation and mechanism understanding. Essential component identification offers great help for understanding its working mechanism. Qualitative detection of EEG oscillations gained much achievement in aiding clinical treatment of epilepsy especially under seizure onset. Potential biomarker of epileptogenic zone includes high-frequency oscillations. Fast activity and gamma (30–100 Hz) interictal epileptiform discharges have close relationship with seizure onset according to intracranial EEG signal analysis [12, 13]. Gamma band activity used to be one significant marker for seizure occurrence [5]. Both fast beta/gamma and slow delta/theta are typical phenomena in focal epilepsies. Low frequency oscillations have been investigated as characteristic EEG patterns that influence seizure onset in temporal lobe epilepsy (TLE) [14]. Higher power and synchronization within theta band (4–7 Hz) and lower power in alpha band (10–13 Hz) demonstrate significant variations in epilepsy patients comparing with healthy controls [15].

Characterized with abnormal synchronized neural firings, the pathological mechanism of epilepsy involves whole brain network rather than certain brain area [16]. Network-based techniques are required for accounting for features of complex systems.

Multiple brain areas and routes involve in seizure course. Besides, the areas and routes vary with specific epilepsy types and patients. Spatiotemporal organization and dynamics research of EEG oscillations are necessary for precise epilepsy evaluation [17]. Basically, topographical EEG analysis quantitatively depicts seizure conditions from temporal and spatial aspect. Under certain conditions, distribution of connection probability follows the rule of power law which signifies that there exist limited brain sites connecting with most other brain areas. Meanwhile, information traffic reshapes corresponding network dynamics such as seizure attacks.

Brain connectome reveals epileptic activity by network analysis [18]. Brain network characteristics unravel functional mechanisms in clinical epilepsy [19]. Many network-based tools have been proposed to depict objective alterations during the process of epilepsy, most of them focused on evaluation indicators including degree, clustering coefficients, the shortest path length, transitivity, and efficiency. Less integrated and more detached networks are demonstrated in children with frontal lobe epilepsy (FLE) particularly when they are cognitively impaired [20]. Moreover, FLE shows more damage in regional efficiency than TLE in structural networks derived from diffusion tensor imaging [21]. Graph theory discovers brain connectivity anomalies effectively [22]. More than four subtypes are divided according to nonlinear analysis of EEG in TLE seizures, and their medial structures demonstrate generic and disorganized network configurations [23]. Asymmetry of instant phase difference is so clear that it was capable of localizing epileptogenic zone [24]. Effects of disease duration go along with network metrics based on machine learning techniques [22]. Lower betweenness centrality reveals the effect of antiepileptic drug use [25]. Connectivity patterns detect propagation of ictal behavior during seizure onset for peeling off epilepsy [26, 27]. It is found that network structure approaches regular type while small-worldness is weakened in epilepsy [28]. In fact, it is known that small world structure produces coherent oscillations accompanied with fast response capability [29]. Although necessary, it is not easy to separate propagation area from epileptogenic zone. How to define epileptogenicity degree requires further study. What is more, not only gamma, but also delta subband EEG oscillations are assumed to be biomarkers for epilepsy, it is not clear what role frequency components play with epileptogenic zone [5]. More efforts should be put into capturing seizure network dynamics due to little consensus on epilepsy network [10].

Link prediction works on probability estimation of future edge generation based on network topology and node attributes. The capability of link prediction is in accordance with the understanding of network organization that illustrated by universal structural consistency index [30]. It is proposed that higher prediction capability corresponds to much more accurate description of network evolution [31]. Node similarity was proposed for solving link generation problem at first. Nodes sharing the same attributes tend to get connected in social networks such as recommendation system. A local naïve Bayes model uncovers hub nodes according to its description of multiple effects from their common neighbors [32]. Essential attributes are defined as node similarity when they are close to each other under certain circumstances [33, 34]. Common neighbor is the most popular structural feature that describes friends' friends. When two nodes share more common neighbors, they are easier to form new edges and the corresponding nodes would gather together into network cluster, just as what happens in social networks [35] and cooperation scientists [36]. Network cluster is an important feature of brain, and evolutional optimization has been adopted for cluster identification of brain networks. Symmetry and matching index has been proposed for depicting cortical connectivity. The combination of network topology and energy deviation is efficient in classifying seizure occurrence in epilepsy patients [37].

In this paper, we propose a novel workflow to investigate seizure dynamics in epilepsy. Link prediction is introduced to reveal alterations in corresponding epileptogenic networks that are determined by experienced clinicians. As a preliminary work, epileptogenic networks are comprised of intrafocus sites and extrafocus sites. Since the advantage of network analysis is its fingerprint depiction of corresponding brains, nine different types of epilepsy covering 20 patients are enrolled in this study. As weight stands for information diffusion or energy cost in the brain, weighted brain networks are derived from preprocessed subband EEG oscillations by way of phase locking value. Time window is applied to generate transient dynamic frequency dependent brain networks. Four popular link prediction algorithms are adopted including CN, RA, AA, and Sorenson algorithms. The performance of link prediction capability is evaluated through AUC (area under the curve). The proposed workflow is tested on long-term EEG recordings covering seizure course. Traditional Granger causality is applied for dynamic information flow detection for comparison. Node clusters are derived by node ranking under link prediction theory. However, these node clusters are gathered together as node sequence first after node ranking. In order to distinguish the roles that played by intrafocus and extrafocus sites, transient variations of node sequences are evaluated by cosine similarity and Euclidean distance. Then node index summation is put forward to get fingerprint evaluation of each patient. The results demonstrate their efficiency in depicting seizure occurrence as well as describing epilepsy types. The layout of the paper is as follows. The materials and datasets are illustrated in detail in Section 2. The techniques and methods are presented in Section 3.  Section 4 demonstrates the experimental results.  Section 5 presents discussions and analyzes of the results. Finally, the conclusions are given in Section 6.

## 2. Materials

### 2.1. Patient Data

The EEG database is accessed from https://epilepsy.uni-freiburg.de/. A total of 8 patients with refractory focal epilepsy are enrolled in the present study. Details of the patients are illustrated in Table 1, where SP = simple partial, CP = complex partial, and GTC = generalized tonic-clonic; H = hippocampal origin and NC = neocortical origin; and d = depth electrode, g = grid electrode, and s = strip electrode. Two patients are frontal lobe epilepsy with hippocampus origin (frontal/H, short to be FLE), four patients are temporal epilepsy with hippocampus origin (temporal/H, short to be TLE), and two patients are temporal, occipital epilepsy with hippocampus origin (temporooccipital/H).

### 2.2. EEG Database

**Figure 1:**
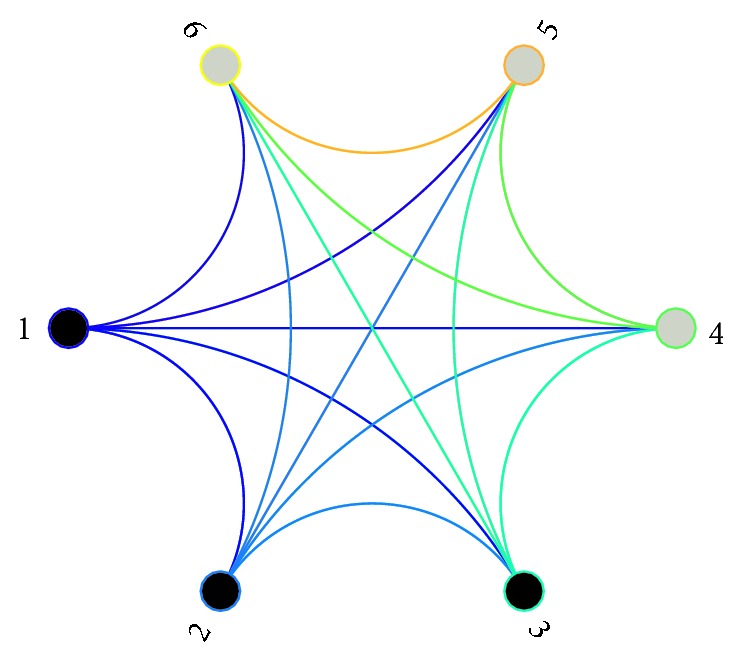
Connection among intrafocus (nodes 1–3) and extrafocus (nodes 4–6) sites in epileptogenic network.

Invasive EEG recording is taken as presurgical epilepsy monitoring at the Epilepsy Center of the University Hospital of Freiburg, Germany. A Neuro-file NT digital video EEG system was applied for recording and EEG signals are sampled at 256 Hz. The recording covers the whole process of seizures. All clinical investigations have been conducted according to the principles expressed in the Declaration of Helsinki. This research was approved by the ethics committee of University Hospital of Freiburg (Ethik-Kommission der Albert-Ludwigs-Universitat Freiburg, Freiburg, Germany), and participants gave written consent for research use of these data [38–42]. Epileptic focus pervades hippocampus, neocortical area of brain structure separately, and these focuses are confirmed by experienced clinicians. These clinicians are also the guidance for the implantation of recording electrodes in the meantime, which ensures the inclusion of both intrafocus and extrafocus areas. Later, the epileptogenic focus and the accurate time for seizure occurrence are identified by experienced surgeon. Long-term EEG signals were recorded by intracranial grid, strip, and depth electrodes. For simplification, intrafocus sites are denoted as 1 to 3 and extrafocus are denoted as 4 to 6 separately as illustrated in Figure 1. It can be assumed that all nodes are connected with other ones except themselves.

Total 30 seizures in 3 epilepsy subtypes in 8 patients are investigated in this study. EEG signals are divided into preictal, ictal, and postictal sessions accordingly. No eye or muscle artifact is recorded in these intracranial EEG signals. Since the strongest noise is 50 Hz power line interference, preliminary EEG preprocessing is comprised of wavelet-based general denoising and 50 Hz power frequency interference elimination. Then signals are removed when their amplitudes exceed 8000 microvoltage. In earlier spectrogram analysis, time-varying power distribution displays in various frequency bands based on short time Fourier Transform. It is found that increased hypersynchronous neural firings occur in a large range of subband EEG oscillations and pervade many areas under seizure attack.

### 2.3. Network Dataset

The network data for algorithm test were obtained from www.linkprediction.org [43], which was adopted many times by researchers in network science for detecting new techniques. It includes six datasets as follows: Jazz musician cooperation network, King James dataset, USair dataset, Adolescent dataset, NetScience dataset, and *C. elegans* dataset. Then simulated scale-free network was brought in together with real EEG network derived from epilepsy patients. The number of nodes for simulated network is 30 while its averaged node degree is set to be close to EEG network. The EEG dataset is downloaded from http://eeg.pl/epi/ for link prediction test where the recording sites will be taken as node number while their interaction will be taken as links [44]. All datasets are divided into train set and test set for algorithm validation. The ratio for train set will not exceed 95 percent.

## 3. Methods

### 3.1. Network Abstraction

**Figure 2:**
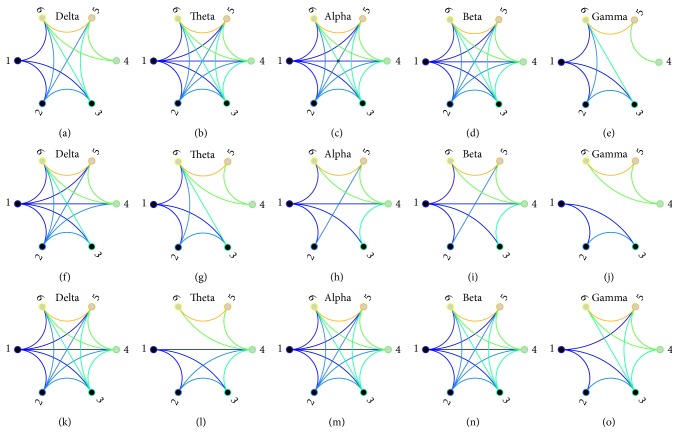
Wiring patterns during seizure course in brain network. Time-varying wiring patterns during seizure course in brain network with TLE, where weighted networks are transformed to be binary at the threshold value of 0.3. Nodes 1 to 3 denote intrafocus electrode sites while nodes 4 to 6 denote extrafocus electrode sites. (a–e) Frequency-dependent wiring links prior to seizure attack derived from delta, theta, alpha, beta, and gamma subband EEG oscillations; (f–j) frequency-dependent wiring links under seizure attack derived from delta, theta, alpha, beta, and gamma subband EEG oscillations; (k–o) frequency-dependent wiring links after seizure attack derived from delta, theta, alpha, beta, gamma subband EEG oscillations.

After preprocessing, EEG signals are divided into five subband oscillations as follows: delta band (0.5–4 Hz), theta band (4–8 Hz), alpha band (8–13 Hz), beta band (13–30 Hz), and gamma band (30–45 Hz). PLV is applied for detecting neural interactions among recording sites [45], where frequency dependent time window is adopted to detect transient fluctuations. Meanwhile, nonoverlapping time window is implemented for getting time variant fluctuations. Network nodes correspond with recording sites individually. All total PLV are considered as weighted connections in corresponding brain areas except that self-connections are eliminated. Higher PLV signifies stronger correlations. The weighted network is assigned to quantify frequency-dependent interaction strength. Wiring pattern is time-varying during the whole process in each subband EEG networks. Since weights stand for interaction strength and energy cost in the brain, binary networks demonstrate stronger connections as weak ties are removed. As shown in Figure 2, distinct changes occur under different situations when the threshold value for the valid link is set to be 0.3. What is more, it is roughly illustrated in Figure 2 that intrafocus and extrafocus sites could be separated from each other in gamma band networks when the connection strength is considered.

### 3.2. Link Prediction Algorithm Comparison

Four algorithms including CN, RA, AA, and Sorenson algorithm are introduced for link predication capability comparison. RA takes node degree into consideration while allocated resource depends on nodes' common neighbors [46]. In the AA technique, the nodes with smaller degree are regarded to have higher contribution. For Sorenson algorithm, the ratio between common neighbor number and its degree summation matters in link production. In order to compare the performance of these algorithms, six real networks are brought in including Jazz musician cooperation network, King James dataset, USair dataset, Adolescent dataset, NetScience dataset, and *C. elegans* dataset [43]. Simulated small-world and scale-free networks are assigned for comparison which is comprised of 30 nodes. Results show that RA technique outperforms other techniques both in real networks and simulated networks just as that reported in the previous literature [46, 47]. RA and local path index is proposed by Tao Zhou and Linyuan [33, 43]. Let *A* (*V*, *E*) denotes a network where *V* = {*v*_1_, *v*_2_, *v*_3_, *v*_*n*_} stands for node set and *E* = {*e*_1_, *e*_2_, *e*_3_, *e*_*m*_} stands for edge set which each edge is connected two separate nodes. Nodes and edges are important components for a network. As node degree counts the number of its neighbors, higher degree might indicate more important role it plays. CN algorithm is the most popular technique for measuring node similarity which is based on the number of common neighbors. Let *φ*(*x*) denotes the set of neighbors of node x and *φ*(*y*) denotes the neighbor set for node *y*, and then node similarity is defined as the number of common neighbors as follows:

(1) $$\begin{array}{c} S_{xy}=\varphi \left(x\right)\;\cap \;\varphi \left(y\right). \end{array}$$

**Figure 3:**
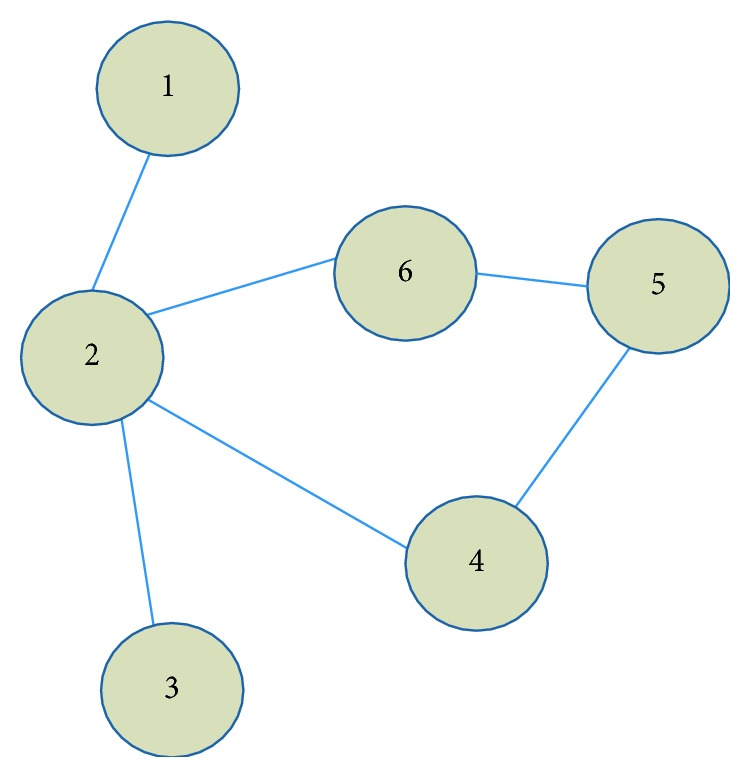
A representative network example.

As illustrated in Figure 3, the similarity between node 2 and node 5 is 2, in other words, *S*_{2, 5}_=2.

RA considers the degree of common neighbors which act as relay station for resource transmission. The definition of RA is as follows:

(2) $$\begin{array}{c} S_{xy}=\sum_{z\in \varphi \left(x\right)\cap \varphi \left(y\right)}\frac{1}{k\left(z\right)}, \end{array}$$

where *φ*(*x*) ∩ *φ*(*y*) denotes the set of common neighbors and *k*(*z*) represents their node degree. Then *S*_{2, 5}_=1 for nodes (2, 5) and *S*_{6, 3}_=1/4 for nodes (3, 6) in Figure 3.

In AA algorithm, it presumes that more contribution could be achieved by the nodes that have smaller node degree. Then, its similarity is defined as follows:

(3) $$\begin{array}{c} S_{xy}=\sum_{z\in \varphi \left(x\right)\cap \varphi \left(y\right)}\frac{1}{\log\;k\left(z\right)}. \end{array}$$

Then the similarity between node 2 and node 5 in Figure 3 would be *S*_{2, 5}_=1/log  2+1/log  2=2.8854.

Sorenson algorithm considers not only common neighbors, but also their degree. The definition is as follows:

(4) $$\begin{array}{c} S_{xy}=\frac{2\times \varphi \left(x\right)\;\cap \;\varphi \left(y\right)}{k\left(x\right)+k\left(y\right)}, \end{array}$$

*k*(*x*) and *k*(*y*) denote node degree for node *x* and node *y*, respectively. Then *S*_{2, 5}_=2/3.

For performance comparison, the edge set *E* will be divided into train set *E*_t_ and test set *E*_p_. The ratio of train set will be increased from 70 percent to 95 percent, and the remaining edges will be taken as test set. Area under the receiver operating characteristic curve (AUC) is adopted for evaluating prediction accuracy. In each test, one existing link would be chosen randomly to compare with the randomly chosen nonexistent edge. Let *n* denotes the total test times, *n*′ denotes the times that the higher value for existed edge than that for nonexistent edge, and *n*″ denotes the times that two values are equal, and then AUC is defined as follows:

(5) $$\begin{array}{c} \text{AUC}=\frac{\left(n^{\prime }+{0.5\;n}^{\prime \prime }\right)}{n}. \end{array}$$

As the value of AUC signifies the accuracy, the higher AUC indicates better performance of the corresponding algorithm. In this study, every algorithm will be tested 100 times to get the finical AUC score.

**Figure 4:**
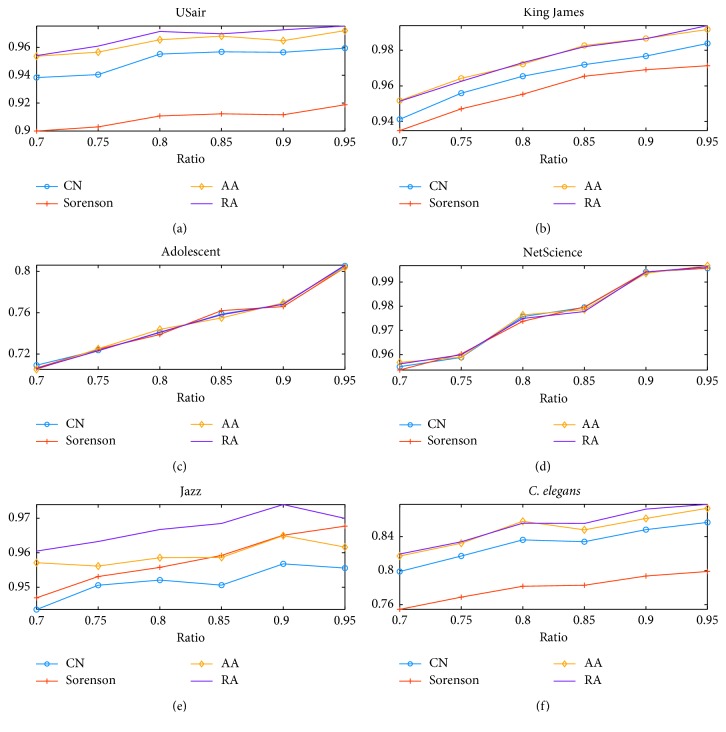
Performance comparison of link prediction algorithms on real networks: (a) USair dataset; (b) King James; (c) Adolescent; (d) NetScience; (e) Jazz; (f) *C. elegans*.

**Figure 5:**
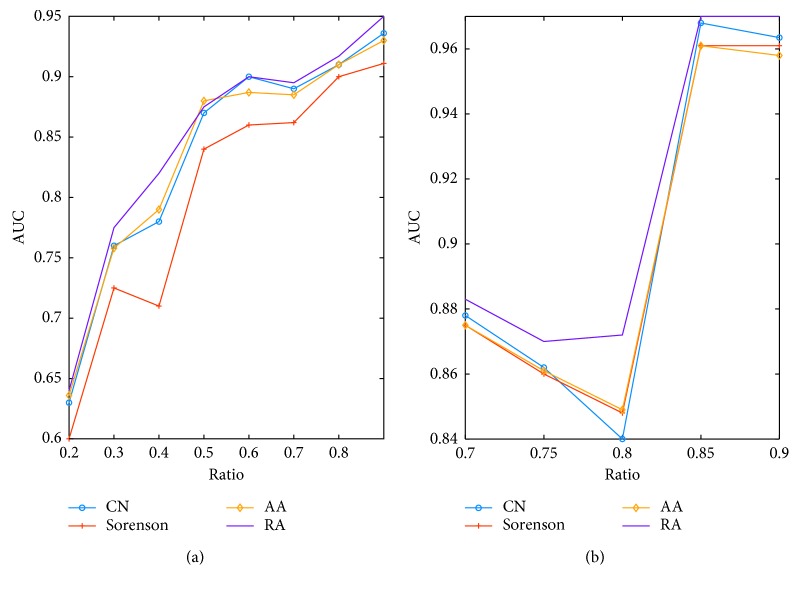
Performance comparison of four link prediction algorithms on two networks: (a) simulated scale-free network; (b) EEG network in alpha band derived from on representative epilepsy patient.

According to our results, it is found out that RA outperforms other techniques as illustrated in Figure 4. In USair connection dataset, King James relationship network, Adolescent friendship network, NetScience scientists, and Jazz musician cooperation networks as well as *C. elegans* structural network, RA outperforms the other three techniques as the train ratio increased from 70 percent to 95 percent. In order to testify its efficiency in small network whose nodes not exceed 30, we brought in simulated scale free network and EEG network derived from http://eeg.pl/epi/ [44]. It turns out that RA is better than other techniques. As illustrated in Figure 5, RA is also better than its candidates in brain networks, and it was adopted for further investigation.

### 3.3. Node Sequence Variation Analysis and Node Index Summation

**Figure 6:**
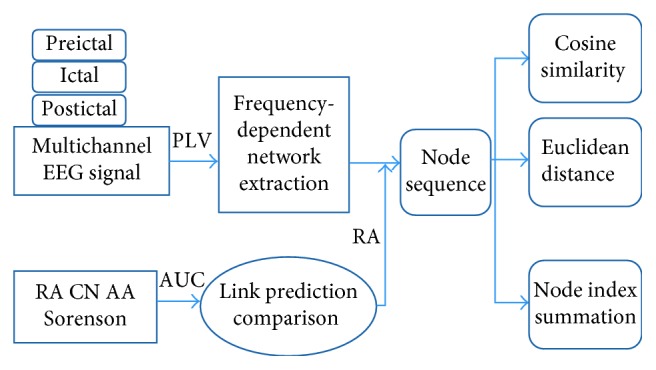
Workflow of dynamic information investigation based on link prediction algorithm.

Transient similarity matrix is derived from the RA technique by ranking nodes according to node similarity. The nodes owning highest similarity are selected as the one in the sequence correspondingly. Then, six nodes that have highest similarity are combined together as node sequence where self-similarity is eliminated. In other words, node sequences are comprised of nodes owning the largest value in similarity matrix produced from RA technique. Cosine similarity and Euclidean distance are taken to measure the variation of transient node sequences, as they could measure the distance between vectors. These sequences are derived from individual adjacency matrix corresponding to preictal, ictal and postictal EEG sessions separately. Since smaller number 1 to 3 indicates intrafocus sites and higher number 4 to 6 indicates extrafocus sites, node index summation is put forward by summing up node sequences to depict the distinct roles that played by intrafocus and extrafocus points. Then node index summation is repeated in 30 seizures of 8 patients with 3 different subtypes with epilepsy. The total workflow is illustrated in Figure 6 as follows.

## 4. Experiment and Results

Time-varying PLV was obtained by nonoverlapping time window on EEG signals. Preictal is set to be 3 minutes prior to occurrence and postictal is set to be 3 minutes after seizure cessation. Initiation and termination of ictal process depends on clinical phenomena and was marked by experienced surgeons. According to spectrogram analysis, it is shown that higher power exist among intrafocus sites than extrafocus ones under seizure onset in TLE patients. Simultaneously, delta band oscillations dominate the energy and gamma band has minimum energy. During the seizure attack, activated nodes could be determined according to spectrogram analysis and node ranking. Besides, sharp increase and decrease of power alteration occur in gamma and delta band EEG oscillations separately.

**Figure 7:**
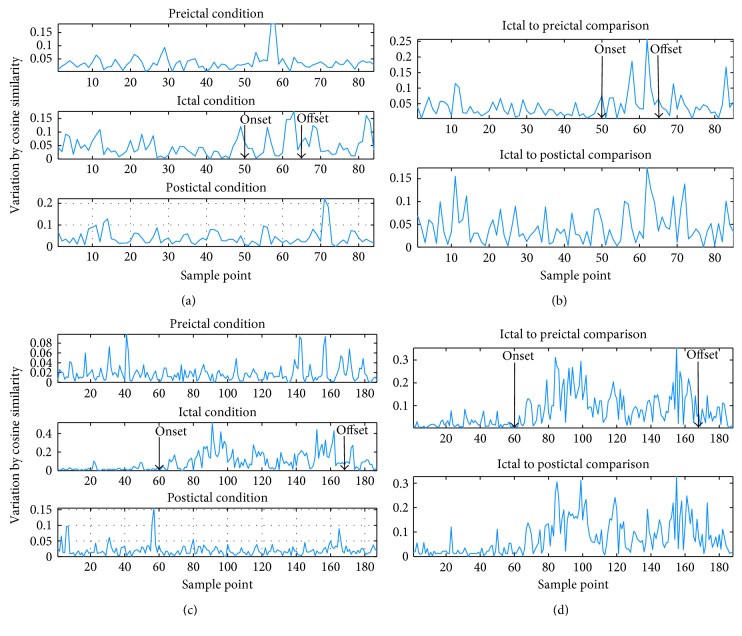
Distance of node sequences during seizure course: (a) alterations vary with time during preictal, ictal, and postictal segments in gamma band EEG networks from one patient with FLE; (b) distance of node sequences when comparing preictal to ictal and ictal to postictal segments in gamma band EEG networks from one patient with FLE; (c) alterations vary with time during preictal, ictal, and postictal segments in gamma band EEG networks from one patient with temporo/occipital epilepsy; (d) distance of node sequences when comparing preictal to ictal and ictal to postictal segments in gamma band EEG networks from one patient with temporo/occipital epilepsy.

**Figure 8:**
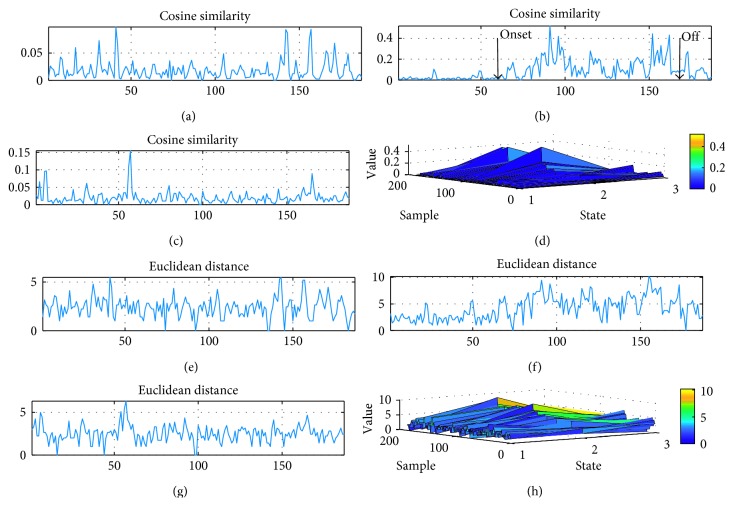
Variation of node sequences during seizure course from a patient with temporo/occipital epilepsy: (a–c) variations detected by cosine similarity under preictal, ictal, and postictal condition separately; (d) variations in preictal, ictal, and postictal condition detected by cosine similarity together; (e–g) variations detected by Euclidean distance under preictal, ictal, and postictal condition separately; (h) variations in preictal, ictal, and postictal condition detected by Euclidean distance together.

**Figure 9:**
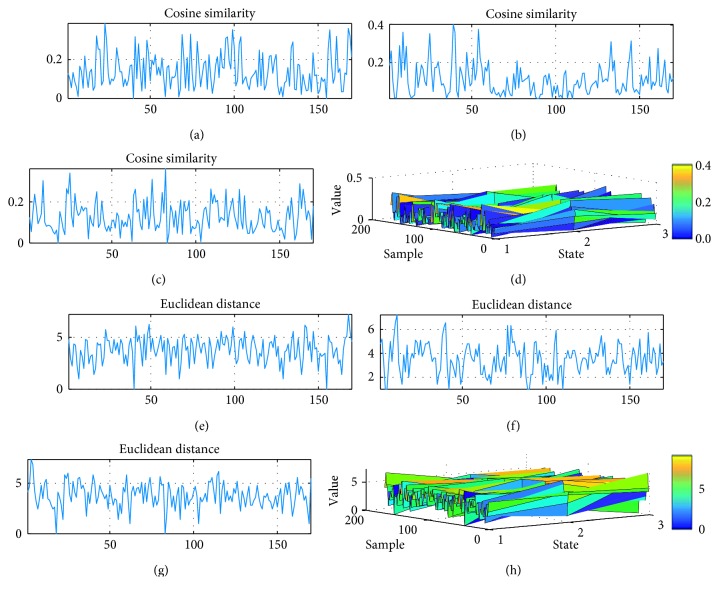
Variation of node sequences during seizure course from a patient with TLE: (a–c) variations detected by cosine similarity under preictal, ictal, and postictal condition separately; (d) variations in preictal, ictal, and postictal condition detected by cosine similarity together; (e–g) variations detected by Euclidean distance under preictal, ictal, and postictal condition separately; (h) variations in preictal, ictal, and postictal condition detected by Euclidean distance together distance of node sequences during seizure course.

Similarity, matrix is obtained by way of RA. Node sequence is produced by combining nodes that correspond with highest value in the similarity matrix. Variation of node sequence is calculated by cosine similarity and Euclidean distance. It seems that they are sensitive to subtypes of epilepsy. As illustrated in Figure 7, from a patient with TLE, fluctuations in node sequences are both time-dependent and frequency-dependent in all EEG networks. It is demonstrated that network organization alters sharply under seizure condition especially in patients with TLE. The variation of node sequences changes strongly compared with that under preictal and postictal condition. Besides, intense network variations are demonstrated both in ictal to preictal and in ictal to postictal comparisons. Specifically, alterations of node sequences could detect seizure onset from gamma band EEG oscillations, while no clear difference are revealed in the other four subband EEG oscillations. The variation trend is in the opposite direction between temporo/occipital and TLE subtypes, which decrease at first and bounce back later during ictal session in the former, and the variation decreases during seizure occurrence with an increase occurs prior to attack in the latter. In temporooccipital/NC subtypes, neither clear decrease nor increase springs out. Figures 8 and 9 display distinct variations of network connections in gamma band in different epilepsy subtypes. Specifically, this time-dependent variation occurs in both temporo/occipital and TLE in descending and ascending directions during the attack process separately.

Specifically, distance of ranked node sequence reflects the time-varying network organizations during seizure process. Besides, the contrary alteration of node sequence variations indicates distinct pathological mechanism of seizures in TLE and temporo/occipital epilepsy. All these attest that link prediction techniques enabling uncovering heterogeneous mechanism of epilepsy, and they might provide new insights for epilepsy investigation.

In order to separate intrafocus from extrafocus sites, nodes are ranked by their similarity according to link prediction analysis. Then, node index summation is presented for characterizing time-varying networks for seizures after node ranking in similarity matrix. Node index summation is calculated in total 8 patients over five subbands. Results demonstrate that under seizure attack condition, the networks tend to be closer to intrafocus. Since 1 to 3 indicates intrafocus sites, higher values of node index summation demonstrate more extrafocus involving in network sequence. As for FLE, more extrafocus sites take part in network sequence in delta band comparing with preictal and postictal conditions. However, more intrafocus focus sites are involved in the other four subbands. In TLE, the roles of intrafocus and extrafocus sites are different as more intrafocus takes charge during seizure process in alpha, beta, and gamma subbands. In temporooccipital epilepsy, intrafocus sites are dominant under ictal condition in alpha, beta, and gamma subbands, while extrafocus sites take charge under seizure attack in delta band, and theta band EEG networks involve more extrafocus sites after seizure termination. As node index summation indicates different roles played by intrafocus and extrafocus sites, the higher value corresponds with more extrafocus sites dominate in the network. It is illustrated that most intrafocus sites take part in network sequence under seizure occurrence. What is more, such tendency is frequency dependent. In gamma band, the networks are more obvious for reflecting seizure occurrence than other subband EEG networks under pathological condition.

We also calculated network metrics on time-varying weighted networks including weighted degree, weighted clustering coefficients, and weighted path length. These measurements could detect the eruption of seizures in the beginning. However, it fails to detect the alteration of network organization during seizure course.

**Figure 10:**
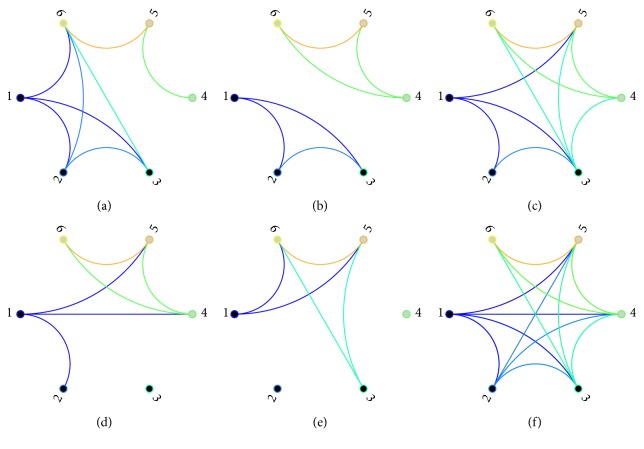
Time-varying wiring patterns in gamma band EEG oscillations: (a–c) binary brain networks for preictal, ictal, and postictal stages at the threshold value of 0.3 in patient with TLE; (d–f) binary brain networks for preictal, ictal, and postictal stages at the threshold value of 0.3 in patient with temporooccipital/H epilepsy.

What is more, we have tested these link prediction techniques on other biological networks including simulated hierarchical and modular networks. It has been found out that RA technique is not applicable for these networks, which might due to their special topology property Figure 10.

## 5. Discussion

Combining link prediction technique with theoretical network topology, we propose a novel workflow for describing and quantifying seizures from intracranial EEG recordings. At first, EEG oscillations are divided into delta (0.5–4 Hz), theta (4–8 Hz), alpha (8–13 Hz), beta (13–30 Hz), and gamma (30–45 Hz) bands separately. A frequency-dependent length of window is brought in to detect transient connection behavior by multichannel EEG signals, whereas time-varying PLVs are derived from these subband EEG oscillations. These PLVs are taken as elements in adjacency matrix. Simultaneously, CN, RA, AA, and Sorenson algorithm are brought in for performance comparison in link prediction based on node similarity. As RA defeats its opponents, the RA technique is adopted to get largest similarity matrix from the weighted networks. Node sequences are detected through these similarity matrixes during seizure condition in nine different kinds of epilepsy. Cosine similarity and Euclidean distance are applied for calculating the variation of node sequence during the total seizure process. Node index summation is put forward for separating intrafocus and extrafocus sites. It is demonstrated that variation in node sequences detect seizure bursts and monitor seizure journey in focal and temporal epilepsy. A majority of networks bounce back to previous condition in these subband EEG oscillations. This might be a reflection of brain plasticity under pathological condition. Take gamma band EEG oscillations as an example; the alteration of wiring patterns increased in temporo/occipital epilepsy with hippocampus origin under seizure onset, while the alterations decreased in TLE with hippocampus origin under the same circumstances. According to node index summation, different roles are played by intrafocus and extrafocus sites during seizure journey. In other words, the involvement is changing of intrafocus and extrafocus sites in the epileptogenic network. What is more, patients' specific information could be obtained by node index summation and frequency dependency appears on epilepsy subtypes.

Normally, social networks are different from biological networks in assortative mixing which makes them easy to percolate and hard to be attacked on high-degree vertex [48]. The powerful capability of link prediction has been testified in social networks. However, the adoption of link prediction is rare in brain networks. Apart from precise localization of the epileptogenic zone, it is important to describe the dynamics in the related cortical area [16–18]. If recording sites are taken as nodes, and their relations are regarded as links, nodes and edges are fundamental elements of the network. Interactions among cortical regions could be illustrated through weighted complex networks. Recurrent seizure occurrence affects brain structure and its function. Tracking seizure dynamics could unravel functional integration and segregation and then aid in clinical treatment. With the advent of complex network, EEG recordings could be investigated for uncovering working mechanisms of the brain integrally and systematically, which reflects faster temporal spontaneous neural oscillations in the brain. Network identification and characterization aids in clinical treatment for medically intractable epilepsy patients [27]. Epileptogenic networks consist focus and other cortical areas involved in seizure initiation and propagation.

In this study, the implantation of recording electrodes covers both intrafocus and extrafocus brain areas. Nodes 1 to 3 denote intrafocus area and nodes 4 to 6 correspond with extrafocus area. Long-term invasive EEG signals are recorded prior to surgery in refractory epilepsy patients. Abnormal neural firings could be eliminated or weakened according to disruption or control of these epileptogenic networks. Previously, experimental results demonstrate that link prediction on weighted network is worse than those on undirected binary network [33]. However, binary links would remove the important message including cooperation strength and energy cost. What is more, such worse results do not appear in our experiment.

Connections among brain regions quantify information flow. Edge weights reflect the similarity distance among brain areas. It is assumed that weak links play important roles in psychiatric pathologies as well as in network function [33]. In other words, weight should be considered as an important component in brain networks. In this work, our real networks contain both binary and weighted ones, and RA technique outperforms other three methods in link prediction. The power of RA technique reflects their general topology property along with network dynamics in these tested networks. Node similarity is an effective indicator for investigating network dynamics [49, 50]. All the adopted four techniques are focused on node similarity. Results demonstrate that this node similarity-based prediction technique could detect seizure attack according to investigating weighted frequency dependent networks.

It is known that brain has to work cooperatively and harmoniously. Disconnected network in brain disorder was proposed by Wernicke in the late 19th century. However, most work focused on quantitative measurement instead of uncovering details. What is more, the small-worldness assumption of brain network requires further investigation [51]. In this paper, we brought in link prediction techniques for describing and quantifying seizures from intracranial EEG recordings. The sliding window enables capturing frequency-dependent network evolution during the course of seizures [52]. Intrafocus and extrafocus sites are denoted as 1 to 3 and 4 to 6 individually. These nodes are ranked to form node sequence according to the largest similarity matrix which was evaluated by RA technique. Nodes that have highest similarity would be combined to form node sequences corresponding to individual recording sites. As an example, the sequence might be 6,54,211 during preictal period and then it changes to 2,21,111 under seizure attack. Cosine similarity is a measure for quantifying distance between vectors and string in machine learning and artificial intelligence. The cosine similarity among these varying node sequences depicts the process of seizures especially in gamma band EEG networks.

It is illustrated that variation of node sequence detects seizure occurrence and monitors seizure process in weighted networks derived from gamma band oscillation. Take TLE as an example; alteration of network organization bursts as soon as seizure occurs in gamma band EEG oscillation, and network disruption is demonstrated in other subband EEG oscillations under attack. Our proposed workflow offers a technique for quantifying network alterations effectively. These various alterations implicit heterogeneity of network evolution accompanied with transient ictal behaviors in distinct epilepsy.

According to our research, RA has the best performance in link prediction based on node similarity. It is assumed that the consideration of the next nearest neighbors enables it to discover transmission and connectivity capability under nonlinear condition [33, 46, 53]. Besides, allocated resource might be better for representing weighted networks. One node's resource could be distributed and flowed to its neighbors, which was determined by network topology, whereas power-law strength-degree correlation produces equilibrium in resource-allocation dynamics [53]. It is postulated that RA has splendid performance in networks owning high clustering coefficients. Brain is such a high-efficient complex system that higher clustering coefficients and longer path length might coexist under pathological epileptic condition. This might contribute to the good performance of the RA technique. As RA has clear physical significance, we could presume that these frequency-dependent varying node sequences and their distances are another reflection of network dynamics in seizures.

## 6. Conclusion

Epileptic seizure investigation from multichannel EEG signals is critical for uncovering the pathology of epilepsy. In this paper, we propose a novel workflow for depicting seizure occurrence in refractory focal epilepsy. This network-based workflow detects the seizure occurrence and monitors the total seizure course, which is the first work aiming at monitoring alteration of network reorganizations throughout seizures as far as we know. The key point is that link prediction technique is adopted to describe connection transitions under seizure attack by way of similarity matrix. It finds out that network variation detecting outburst as well as termination of seizures according to gamma band EEG oscillations in frontal and temporal focal epilepsy.

Weighted dynamic network organization reveals information transmission and interaction strength during seizure course. Results demonstrate that our workflow takes the picture of network connection transitions all the way to seizure when compared with ordinary network metrics including weighted clustering coefficients. In the beginning, four link prediction techniques are brought in for performance comparison including CN, RA, AA, and Sorenson algorithm which have demonstrated their powerful capability in social networks based on evaluating local property of networks. It is supposed that higher predictability corresponds with better description of network dynamics. Since RA is superior to other three rivals, node sequences are produced from similarity matrix by ranking nodes based on RA technique. Node sequence variations are calculated covering seizure onset in subband EEG networks in all subtypes. Cosine similarity is better than Euclidean distance in detecting variance in these node sequences during the whole seizure process. Experimental results reveal that it could quantitatively characterize and monitor seizure occurrence. Distinct variations in node sequences are demonstrated in various epilepsy subtypes. These variations might be a reflection of their heterogeneous pathology. Node index summation quantifies both temporal and frequency information about subtype epilepsy. In conclusion, link prediction might be an efficient assistant technique for uncovering brain network dynamics in epilepsy.

The presented workflow introduces link prediction technique for depicting brain networks. Epileptogenic cortical network plays dominant roles in working mechanism of epilepsy. Heterogeneity is a critical problem when investigating epilepsy's pathology. The combination with link prediction and network organization offers new insights for understanding epilepsy through the point of seizure occurrence. In refractory focal epilepsy, surgery is a good choice to ease the pain of patients. And the separation of intrafocus and extrafocus sites aids in diagnosis and clinical treatment in epilepsy research. According to our experimental results, RA ranks individual nodes efficiently while variation in node sequences detects seizure occurrence. Limited by the number of subjects in this research, our preliminary work did not get statistical results for characterizing distinct epileptic brain networks. Therefore, more research is needed for deeper understanding. For example, more investigation should be taken to uncover the contrary changes between TLE and temporo/occipital epilepsy brain especially in gamma band EEG oscillations. What is more, RA should be investigated further by computational neuroscience methods. Besides, as gamma band EEG oscillation is believed to play important role in higher cognitive function; distinguishable alteration in gamma band networks needs more attention in epilepsy research.

## Figures and Tables

**Table 1:** Detailed information of patients.

Patient	Sex	Age	Seizure type	H/NC	Origin	Electrodes	Seizures analyzed
1	F	15	SP, CP	NC	Frontal	g, s	4
2	M	38	SP, CP, GTC	H	Temporal	D	3
3	F	26	SP, CP, GTC	H	Temporal	d, g, s	5
4	F	16	SP, CP, GTC	NC	Frontal	g, s	5
5	F	31	CP, GTC	H	Temporo/occipital	d, g, s	3
6	F	42	SP, CP, GTC	H	Temporal	D	3
7	M	47	SP, CP, GTC	H	Temporal	D	5
8	F	22	SP, CP, GTC	H	Temporo/occipital	d, s	2
